# Double strand RNA delivery system for plant-sap-feeding insects

**DOI:** 10.1371/journal.pone.0171861

**Published:** 2017-02-09

**Authors:** Saikat Kumar B. Ghosh, Wayne B. Hunter, Alexis L. Park, Dawn E. Gundersen-Rindal

**Affiliations:** 1 United States Department of Agriculture, Agricultural Research Service, Invasive Insect Biocontrol and Behavior Laboratory, Beltsville, Maryland, United States of America; 2 United States Department of Agriculture, Agricultural Research Service, Horticultural Research Laboratory, Fort Pierce, Florida, United States of America; Natural Resources Canada, CANADA

## Abstract

Double-stranded RNA (dsRNA)-mediated gene silencing, also known as RNA interference (RNAi), has been a breakthrough technology for functional genomic studies and represents a potential tool for the management of insect pests. Since the inception of RNAi numerous studies documented successful introduction of exogenously synthesized dsRNA or siRNA into an organism triggering highly efficient gene silencing through the degradation of endogenous RNA homologous to the presented siRNA. Managing hemipteran insect pests, especially *Halyomorpha halys* (Stål) (Heteroptera: Pentatomidae), the brown marmorated stink bug (BMSB), is critical to food productivity. BMSB was recently introduced into North America where it is both an invasive agricultural pest of high value specialty, row, and staple crops, as well as an indoor nuisance pest. RNAi technology may serve as a viable tool to manage this voracious pest, but delivery of dsRNA to piercing-sucking insects has posed a tremendous challenge. Effective and practical use of RNAi as molecular biopesticides for biocontrol of insects like BMSB in the environment requires that dsRNAs be delivered *in vivo* through ingestion. Therefore, the key challenge for molecular biologists in developing insect-specific molecular biopesticides is to find effective and reliable methods for practical delivery of stable dsRNAs such as through oral ingestion. Here demonstrated is a reliable delivery system of effective insect-specific dsRNAs through oral feeding through a new delivery system to induce a significant decrease in expression of targeted genes such as JHAMT and Vg. This state-of-the-art delivery method overcomes environmental delivery challenges so that RNAi is induced through insect-specific dsRNAs orally delivered to hemipteran and other insect pests.

## Introduction

Insect pests around the world are the most extensive group of animals adversely affecting urban and rural plants and other animals. *Halyomorpha halys* (Heteroptera: Pentatomidae), the brown marmorated stink bug (BMSB), is an invasive insect pest, which poses a significant ecological and economic constraint of billions of dollars collectively. In 1989, this new invasive insect pest from Asia (China, Taiwan, Korea, and Japan) was accidentally introduced into Allentown, PA [1]. BMSB is a polyphagous piercing/sucking feeder damaging over 300 known plant hosts including specialty crops such as apples, stone and pome fruits, grapes, ornamental plants, vegetables, seed crops, as well as such staple crops as soybean and corn. BMSB has been detected in 43 states in the United States predominantly in the Mid-Atlantic region (DE, MD, PA, NJ, VA, and WV) as well as in Canada and Europe [2]. Along with crop damage BMSB can elicit allergic reactions leading to conjunctivitis and rhinitis in individuals sensitive to aeroallergens or contact dermatitis upon exposure to the crushed animal [3,4]. This invasive insect pest is also attracted late in the year in large numbers to structures such as houses, schools and other indoor spaces that provide a safe overwintering spot until spring for mating and egg laying [2].

RNA interference (RNAi) is a well described gene regulatory mechanism wherein exogenous dsRNA is introduced into the cells of eukaryotic organisms and targets degradation of host cell mRNAs containing sequences complementary to the dsRNA [5]. RNAi depletes host mRNA either by transcriptional gene silencing or at a posttranscriptional level thereby modulating the level a protein is produced [6]. The RNAi pathway involves cleaving the dsRNA by Dicer within cells. Dicer is a member of the RNase III superfamily of bidentate nucleases that are evolutionarily conserved in worms, flies, plants, fungi and mammals [7–9]. The resulting 19–21 base pair, short RNAs or siRNAs, next are unzipped and one strand, referred to as the guide strand is incorporated into the RNA-induced silencing complex (RISC). The RISC-RNA complex in combination with Argonaute, a multi-domain protein containing an RNAse H like domain, degrades the corresponding mRNA thereby reducing protein expression [10,11].

Double stranded RNA, dsRNA, was first introduced into *Caenorhabditis elegans* by way of microinjection by Fire and Mello [12] and shown to reduce the *unc-22* gene expression. Then another report demonstrated RNAi using microinjection in *Drosophila melanogaster* to ‘silence’ the expression of frizzled genes [13]. Subsequently, effective RNAi has been reported where dsRNA was delivered by microinjection. In aphids, dsRNA was dorsally injected in the middle of L3 abdomen of immobilized pea aphid (*Acyrthosiphon pisum*) for calreticulin and cathepsin-L [14], and a target salivary gland transcript [15] knockdown; while in honey bee (*Apis mellifera*) injection was made dorsally between the 5^th^ and 6^th^ abdominal segment and to eggs for vitellogenin transcript depletion [16]. Araujo and colleagues noted that dsRNA delivery by microinjection or ingestion to nymphs of the hemipteran triatomine bug (*Rhodnius prolixus*) showed depletion of proteins from the *nitrophorin 2* gene. But dsRNA delivered by ingestion was less traumatic and these insects remained healthier than their injected counterparts [17,18]. Non-sterile septic punctures have been shown to elicit increased expression of immune- related genes in BMSB [19]. Delivery of dsRNAs by injection is not only tedious and impracticable for developing a successful biopesticide, but may also induce mortality due to trauma at the site of injection rather than to RNAi.

Mello and colleagues induced the *pos-1* embryonic lethal phenotype in the F1 progeny of *C*. *elegans* by simply soaking the worms in dsRNA to induce specific interference [12]. Another study demonstrated RNAi by delivering *gus*-dsRNA to *D*. *melanogaster* neonates by a similar soaking method using a solution containing species-specific dsRNA. Interestingly, mortality across four different species of *Drosophila* was reported when dsRNA was delivered by feeding *tubulin-*dsRNA to these animals while species-specific insecticidal effects were reported when *vATPase*-dsRNA was orally-delivered to different insect species including flour beetle (*Tribolium castaneum*), pea aphid (*A*. *pisum*) and tobacco hornworm (*Manduca sexta*) [20]. Another study successfully demonstrated depletion of expression of multiple genes in potato/tomato psyllid (*Bactericerca cockerelli*) using an artificial diet facilitated delivery protocol [18]. Exogenous application of dsRNA including topical application and root treatments to soil of potted plants, field trees and grapevines [21,22], along with successful use of plant cuttings which absorbs dsRNA for feeding bioassays have been shown to work for some hemipterans, like leafhoppers (*Homalodisca vitripennis*), psyllids (*Diaphorina citri and B*. *cockerelli*) [21,23] and planthopper (*Nilaparvata lugens*) [24].

One of the first bioassays inducing RNAi through oral ingestion of dsRNA in a chewing insect was demonstrated in the Western corn rootworm (WCR) (*Diabrotica virgifera virgifera*). WCR-specific dsRNA was applied to WCR artificial diet containing agar for feeding the insects [25]. Numerous dsRNAs were identified that depleted the expression of specific genes resulting in larval stunting as well as mortality [*ibid*]. Another novel method of delivering dsRNA was demonstrated through nanoparticle-mediated depletion of target RNA in mosquitoes. Chitosan was used to produce stable chitosan-dsRNA nanoparticles through electrostatic interaction and delivered to mosquitoes through artificial diet for successful RNAi [26]. Successful oral delivery of dsRNA into insects via ingestion has been achieved for many insects: through artificial or natural diets (reviewed in [27]); by droplet feeding in light brown apple moth (*Epiphyas postvittana*) [28], diamondback moth (*Plutella xylostella*) larvae [29], and honey bees [30,31]; through blood meal in tsetse fly (*Glossina morsitans morsitans*) [32]; by dsRNA-soaked paper disks in termite (*Reticulitermes flavipes*) [33]; and through dsRNA-sprayed leaves in weevils [23] to name a few. Even so, development of effective RNAi strategies in insects are challenging to produce [34–36] and some insect taxa are highly variable in response to ingested dsRNA [37], including lepidopterans [34,38], some hemipterans [39,40], coleopterans [27,41,42], and locusts [43].

One of the most difficult insect groups to target with this technology has been sap or phloem feeding hemipteran insects, since delivery of dsRNA must be into vascular tissues where they feed. Whole plants (citrus and grapevines) were treated with exogenous dsRNA which was delivered to psyllid and leafhoppers, by root drench, foliar sprays, trunk injections, or absorption by cuttings [21,22,44] along with several reviews on possible RNAi strategies in plant-feeding Hemiptera [40]. Transgenic plants expressing species-specific dsRNA were used to silence genes in the cotton bollworm and WCR indicating a steady progress towards RNAi technology [25,45]. In the current study, delivery of nutrients for rearing of BMSB through a natural diet exceeded was superior to that using artificial diet. Consequently the successful delivery of exogenously synthesized dsRNA to the BMSB through a vegetable delivery system and significant induction of the RNAi mechanism was demonstrated for the first time using an oral delivery method. The delivery of gene-specific dsRNAs such as Juvenile hormone acid O-methyltransferase (JHAMT) and vitellogenin (Vg) using this newly developed vegetable delivery system also induced RNAi in BMSB nymphs. The new method supplants conventional RNA delivery and RNAi systems that used topical sprays of dsRNA for plant feeding insects by using their natural plant host. The use of highly attractive vegetables, developing fruits, or native fruiting structures which are not used for human consumption, may be used for delivery of dsRNA, which is of particular importance for the continued development of biopesticide pest and pathogen management.

## Results

### Vegetable mediated delivery of treatment

**Fig 1:**
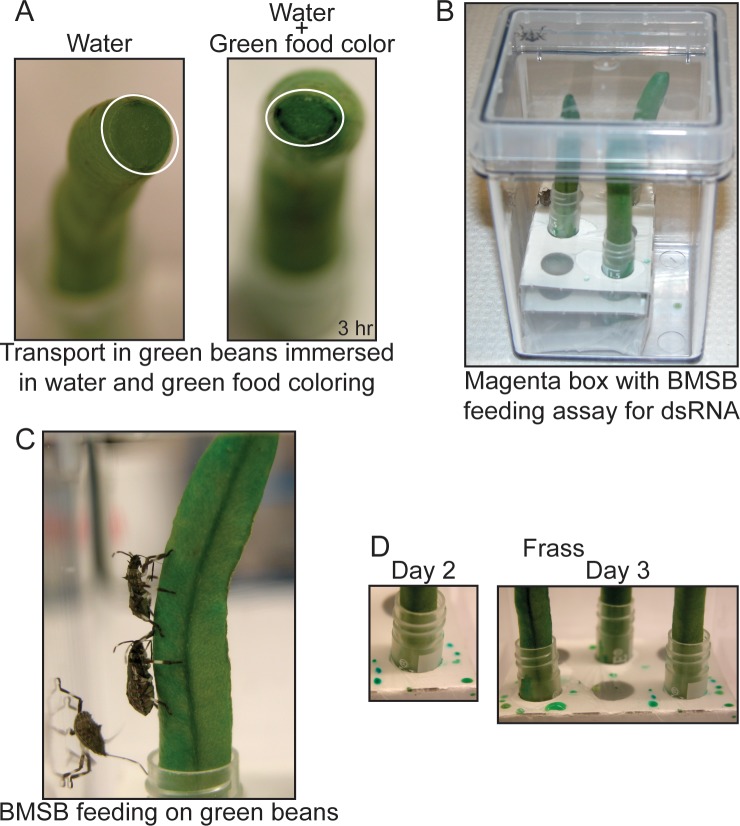
Delivery of nutrients through green beans. (A) Certified organic green beans were immersed in ddH_2_O or ddH_2_O solution with green food coloring for a period of 3 hrs. Transport of green food coloring was observed in the green bean encircled by the oval area at the exposed calyx. (B) BMSB feeding bioassay. 3 animals were placed in a magenta jar with 3 greens beans immersed in either 2 ml microcentrifuge tube containing ddH_2_O or a solution of ddH_2_O and green food coloring. (C) BMSB placed in magenta jars are able to pierce through the green beans and reach the diet with their stylets. (D) BMSB excreta observed on day 2 and 3 post ingesting a solution of ddH_2_O and green food coloring through green beans.

To develop an oral delivery approach for biocontrol using RNAi in the invasive insect pest BMSB, we developed and tested a vegetable delivery method. Thin organic green beans; *Phaseolus vulgaris* L., were selected as the medium for delivery of dsRNA or other treatments to the animal. BMSB feeds on this cultivar crop by piercing into the vascular tissue using their needlelike stylets. The BMSB feeds by alternate salivation and ingestion with slow movement of stylets in a lacerate-and-flush feeding method causing considerable damage to the cultivar crops [46]. We took advantage of this feeding style by testing the delivery via green bean of green food coloring as compared to water. A solution of green food coloring was mixed at 1:10 ratio with water to imitate dsRNA. Slender green beans were trimmed from the calyx, inverted and immersed into either the food coloring solution or water in a 2 ml microcentrifuge tube. Due to flow of phloem and capillary action, the solution reached the style of the bean through the vascular tissue. This was indicated by the green coloration of the peripheral vascular tissue at the style (Fig 1A). A total of three beans were placed in magenta vessels and groups of 3 animals were treated per vessel (Fig 1B).

Early fourth instars were selected originating from the same egg mass and starved for 24 hrs before resuming feeding. The animals fed on the upright green beans by inserting their stylets into the vascular tissues (Fig 1C). Successful feeding on the bean with green food coloring was evidenced by green excreta, which indicated the material had been delivered orally and had passed through the gut before excretion. Green excreta droplets were observed on day 2 post feeding, visualized as green dots, and further increased in content on the third day (Fig 1D).

### Selection of a suitable diet

**Fig 2:**
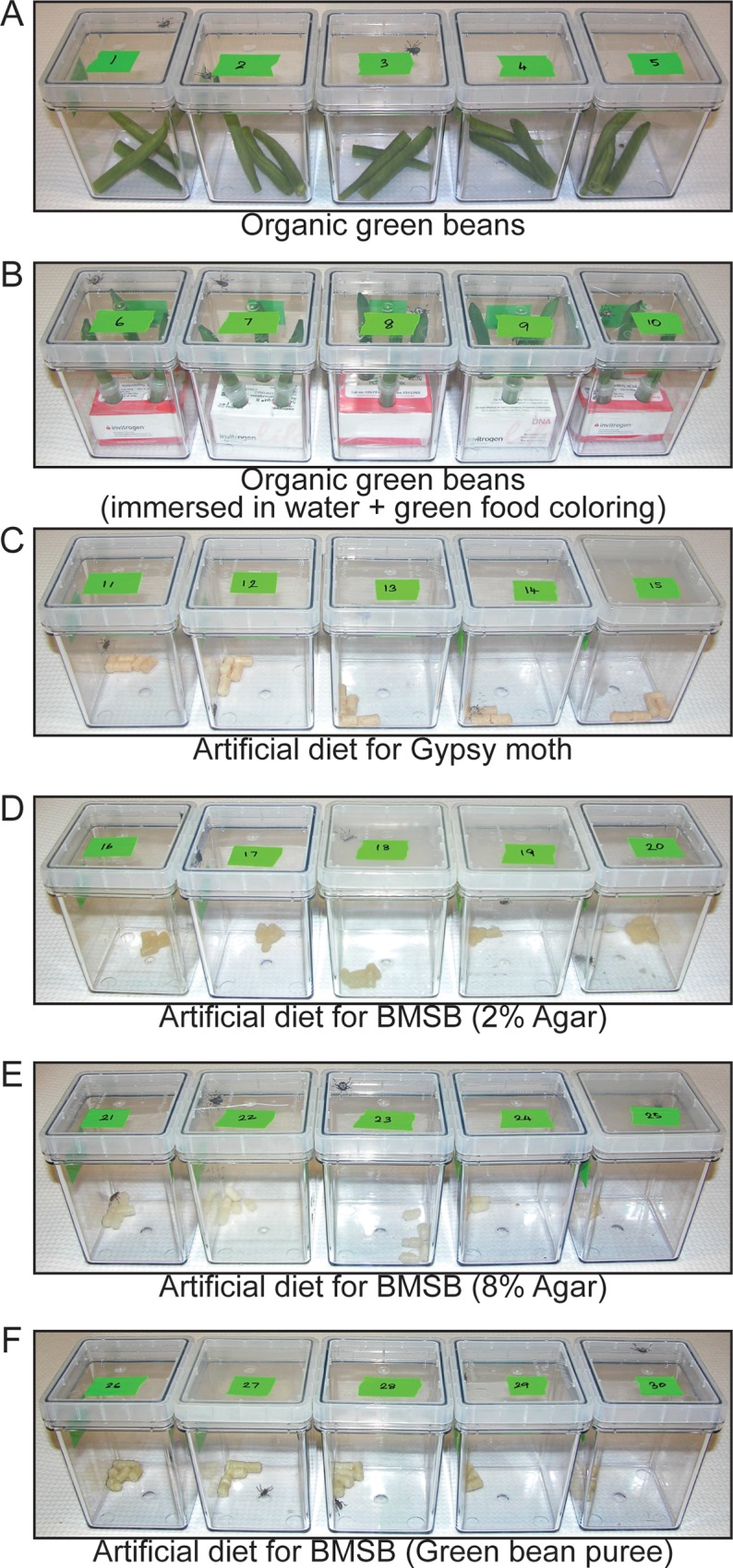
Comparison of natural and artificial diets. Six diets were compared to assess optimal rearing of BMSB. Each magenta jar contained one BMSB and test diet as follows; (A) BMSB reared on green beans, (B) BMSB reared on green beans immersed in 300 μl of ddH_2_O, (C) BMSB reared on artificial gypsy moth diet, (D) BMSB reared on artificial diet formulated for BMSB consisting of applesauce and 2% agar, (E) BMSB reared on artificial diet formulated for BMSB consisting of applesauce and 8% agar and, (F) BMSB reared on artificial diet formulated for BMSB consisting of green bean puree.

Next, the feasibility of an optimal artificial diet was tested and compared to vegetable diet for delivery of dsRNA. Untreated organic green beans were used as a control while green beans immersed in water were used as a new delivery method (Fig 2A & 2B). Because there was precedence for dsRNA delivery to insects through artificial diets, four different artificial diets were prepared containing various ingredients (Fig 2C–2F). These diets contained ingredients that would be attractive to BMSB consisting of applesauce and apple juice (Fig 2D & 2E) or green bean puree (Fig 2F) and an artificial gypsy moth diet used to raise gypsy moth colonies (Fig 2C) (Bell et al. 1981, pg 599 in [47]). Artificial diets were prepared and freeze dried so the treatment could be delivered using a solution that was used to rehydrate the pellets without disintegration of the diet.

**Fig 3:**
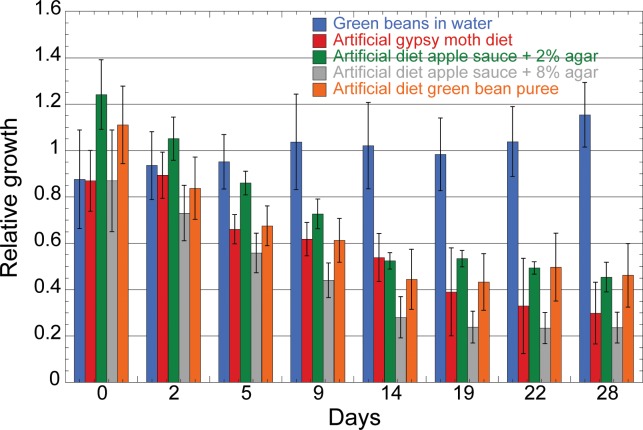
Effect of various diets on BMSB nymph growth. BMSB nymphs 5 each were allowed to feed for a period of 4 weeks during which their body masses were recorded. Diets consisting of green beans; green beans immersed in water; artificial diet for gypsy moth; artificial diet of applesauce and 2% agar; artificial diet of applesauce and 8% agar and, artificial diet consisting of green bean puree. The plot has been normalized to the control diet of green beans. Data was expressed as mean ± SEM. A one way analysis of variance (ANOVA) was performed to test for statistical significance of data, P-value of 0.00048.

The animals were allowed to feed on these diets for a period of 4 weeks and monitored for any physiological changes. The diets were changed every 3 days and replenished with new diet. The animals were observed as individuals and their physiological conditions were recorded. The animals feeding on artificial diet showed notable survival. Although the animals had similar body masses until day 2 of feeding as compared to the diet of green beans immersed in water, by day 22 the body masses of animals feeding on artificial gypsy moth diet decreased by 60 percent with 40 percent survival (Fig 3). Body masses of insects fed on the other diets consisting of applesauce or green bean puree also displayed a 60 percent decrease with a significantly lower mortality than animals feeding on artificial gypsy moth diet (Fig 3). This seems indicative of lower dietary intake in animals feeding on the artificial diets. Animals fed on green beans immersed in water displayed no mortality and a stable increase in body mass (Fig 3) indicating this was a superior method for oral delivery of dsRNA.

### Mobility of *in vitro* transcribed dsRNA through green bean

**Fig 4:**
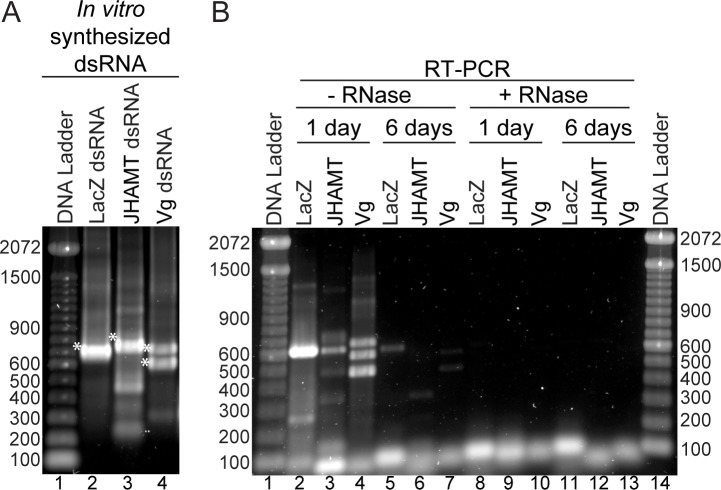
Analysis of dsRNA delivered through green beans. (A) dsRNA of LacZ gene (lane 2), JHAMT (lane 3), and Vg (lane 4), were obtained after PCR products from genomic DNA were amplified with primers containing T7 promoter sequence. These fragments were further transcribed using T7 RNA polymerase; the obtained *in vitro* transcribed dsRNA was confirmed by electrophoresis on 1% agarose and visualized by staining with Sybr Gold® (Life technologies) alongside a DNA ladder (Lane 1). (B) Total RNA extracted from green beans immersed in 5 μg of dsRNA for 1 day (Lanes 2–4) or 6 days (Lanes 5–7) was subjected to cDNA synthesis. 2 μg of this total RNA was subjected to RNase A digestion (0.5 μg/μl final) for 1 hr (Lanes 8–13). RT-PCR was performed on representative cDNAs of LacZ, JHAMT and Vg genes following electrophoresis on a 1% agarose gel and visualized by staining with Sybr Gold (Life technologies) (Lanes 2–13) alongside a DNA ladder (Lanes 1 and 15).

The mobility of green food coloring through green bean vascular tissue showed that when the bean was immersed upright in a solution it was carried against gravity to the bean style. We tested whether this phenomenon occurred while using dsRNA and whether the nucleic acid was stable when delivered through the bean vascular tissue. *In vitro* synthesized dsRNAs for BMSB-specific genes; JHAMT and vitellogenin (Vg) were selected for RNAi while LacZ, a gene encoding ß-galactosidase amplified from *Escherichia coli* genomic DNA was used as negative control (mock) (Fig 4A). The beans were immersed for 24 hrs in 0.017 μg/μl of dsRNA at concentrations of 0.67 μg/cm length of green bean. Subsequently, 0.5 cm of the style region was clipped followed by excision of a 1 cm region from the bean to be tested. Total RNA isolated from the beans immersed in dsRNA was analyzed by RT-PCR using gene specific oligonucleotides. Results indicated that dsRNA introduced into the beans for delivery was stable when compared with dsRNA synthesized prior to its uptake in the bean (Fig 4A compare lanes 2–4 to 4B lanes 2–4). Discrete bands in dsRNA lanes of JHAMT and Vg were representative of homologs or isoforms of these genes as confirmed by DNA sequencing the PCR products (data not shown; NCBI accession numbers and primers in S1 Table). Small minor bands may have been a result of either degradation products or premature termination by T7 RNA polymerase. Overall this demonstrated that BMSB-specific dsRNA was successfully transported through the bean vascular tissues to the style.

In addition to the stability of dsRNA delivered through green bean, the persistence of dsRNA was of a crucial importance especially for agricultural crops. Dubelman and colleagues successfully demonstrated that dsRNA was degraded and biologically inactive in soil after a period of 36 hours indicating accumulation or persistence of dsRNA in the environment was unlikely [48]. To assess the persistence of dsRNA a study was performed to validate its degradation in green beans. The beans were immersed in 0.017 μg/μl of dsRNA for 6 days and total RNA was isolated and analyzed by RT-PCR. Results indicated that persistence of dsRNA after 6 days was greatly diminished (Fig 4B, lanes 2–4 compared to 5–7). To test if the bands visualized on the gel were actually resultant of dsRNA the beans were immersed in, a part of the total RNA isolated was subjected to RNase A treatment prior to cDNA synthesis. No similar sized bands were observed when the RT-PCR reaction was separated by gel electrophoresis (Fig 4B, lanes 8–13 compared to lanes 2–7), denoting that the bands in lanes 2–7 were representative of dsRNA absorbed by the beans. Some discrete bands observed may be non-specific amplification by RT-PCR. Complete degradation in plant cells, which have absorbed dsRNA by dsRNA specific ribonucleases varies due to the concentration and localization of the dsRNA to the vacuole [49]. Since vascular tissues, xylem and phloem may provide temporary sites with reduced enzyme activity where dsRNA may reside safely until it enters an animal cell and induces the RNAi mechanism.

### RNAi in BMSB using green bean mediated dsRNA delivery

**Fig 5:**
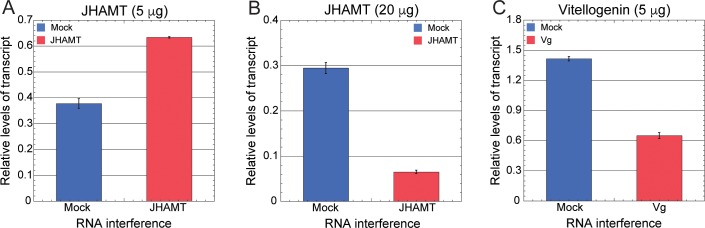
Quantitative RT-PCR analysis of transcript levels after RNAi-mediated depletion of genes in BMSB. Total RNA from 3 individual BMSB 4^th^ instar nymphs fed on JHAMT (A) 5μg (0.017 μg/μl), (B) 20μg (0.067 μg/μl) and *Vg* (C) 5μg (0.017 μg/μl) dsRNAs in 300 μl of ddH_2_O delivered through green beans was isolated and the levels of transcripts were measured by qPCR. LacZ RNAi (Mock) served as a negative control. The 18s RNA was used as an internal standard to correct for differences in RNA recovery from tissues. Results are from three biological replicates, and error bars indicate SEM. A one way analysis of variance (ANOVA) was performed to test for statistical significance of data that indicate significant differences at P < 0.0001 level.

Next we investigated if RNAi could be successfully achieved to deplete specific genes in the invasive insect pest, BMSB through feeding. We demonstrated above that dsRNA was stably transported by green beans and then posited this may be delivered efficiently to BMSB for targeted gene depletion. This is particularly important because most success with RNAi in hemipteran insects has been induced via microinjection of dsRNA [14, 50]. To test this hypothesis, beans were immersed in a solution of either 0.017 μg/μl or 0.067 μg/μl of *in vitro* synthesized dsRNA for BMSB JHAMT and Vg. Another set consisting of beans immersed in water alone or LacZ dsRNA (Mock) was also used as controls. Three BMSB 4^th^ instar nymphs were allowed to feed on green beans in a magenta vessel as described above (Fig 2) for a period of five days. The levels of gene expression were evaluated using qPCR for three biological replicates compared to an internal control. Observations revealed that when the animals were allowed to feed on beans immersed in 0.017 μg/μl solution of dsRNA of JHAMT and Vg only the level of Vg transcript was significantly depleted *in vivo* by approximately 2.2-fold (Fig 5C). Interestingly, when the animals were fed on beans immersed in a solution containing 0.067 μg/μl of JHAMT dsRNA, a considerable 4.5-fold decrease in the level of *JHAMT* expression was observed (Fig 5, compare panels A and B). Using this innovative delivery system, RNAi screens in BMSB have been further evaluated to study effect(s) on transcript levels. These screens included the systematic analysis of many biological processes and biosynthetic pathways in the animal (Ghosh and Gundersen-Rindal, submitted). These results indicate RNAi mediated gene silencing can be accomplished efficiently using the vegetable delivery protocol. We also infer that using this method can be used to deliver the concentration of required dsRNA can be delivered in a dose dependent manner for an effective RNAi.

### Vegetable mediated delivery in other hemipteran insects

**Fig 6:**
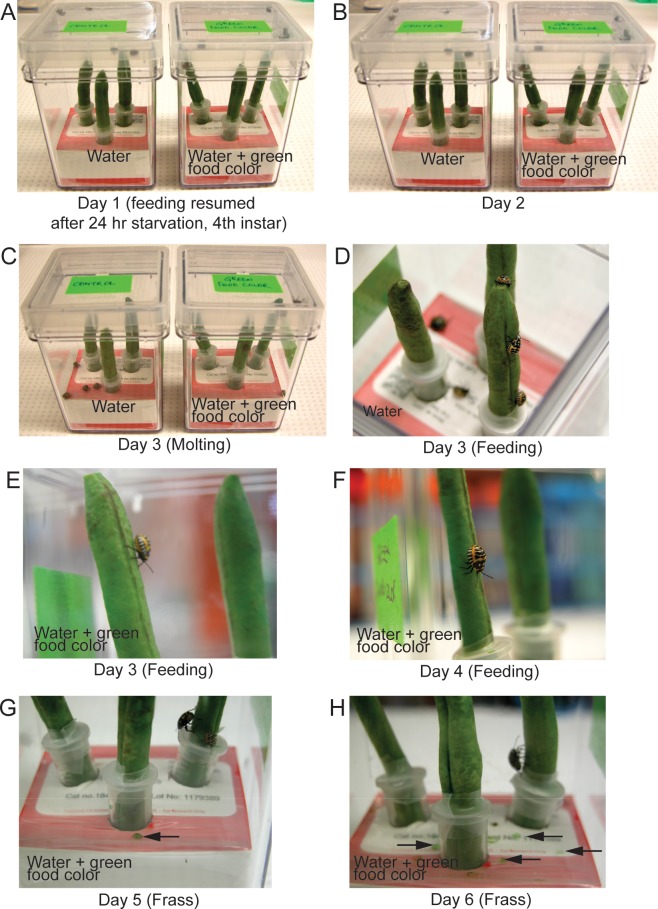
Harlequin bug (*M*. *histrionica*) feeding on green beans. (A) Certified organic green beans were immersed in ddH_2_O or ddH_2_O solution with green food coloring for a period of 3 hrs. 3 each of 4^th^ instar HB were allowed to resume feeding on these beans after 24 hr starvation in each magenta vessel. (B) HB feeding bioassay day 2. (C) & (D) Some animals were observed to molt on day 3 of feeding. (E) & (F) Animals were observed to be feeding on green beans on days 3 and 4 respectively. (G) & (H) Green colored excreta was observed on day 5 and 6 of feeding HB with green beans immersed in solution of water and green food coloring.

**Fig 7:**
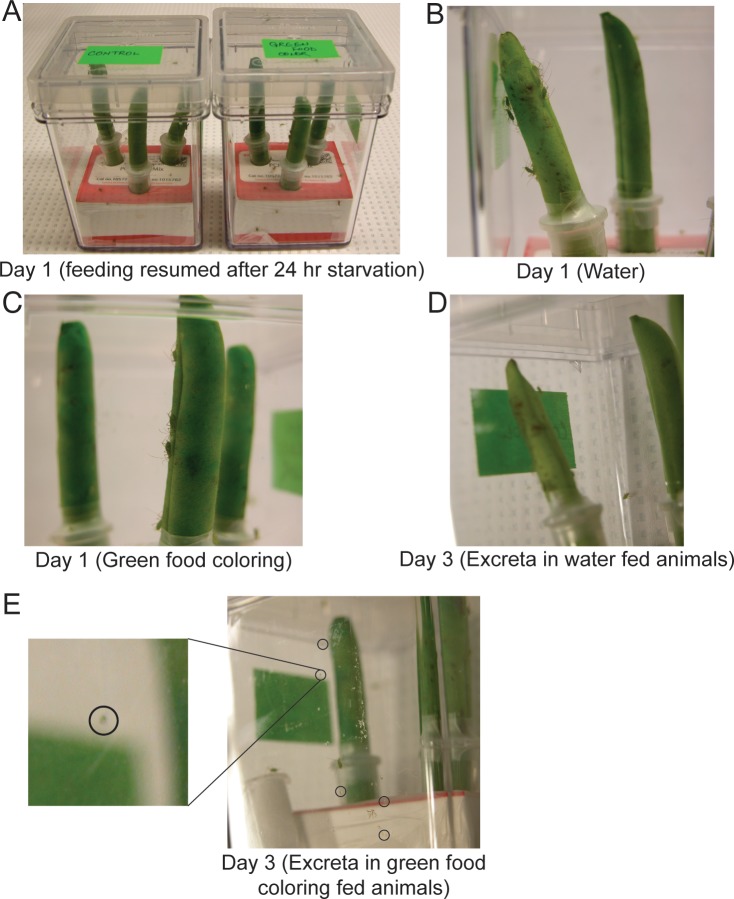
Pea aphid (*A*. *pisum*) feeding on green beans. (A) Certified organic green beans were immersed in ddH_2_O or ddH_2_O solution with green food coloring for a period of 3 hrs. 15 animals each of pea aphids were allowed to resume feeding on these beans after a 24 hr starvation. (B) & (C) pea aphid feeding bioassay. Animals feeding on beans immersed in either ddH_2_O or a solution of ddH_2_O and green food coloring respectively. (D) Excreta droplets were barely observed on day 3 of feeding pea aphids with green beans immersed in water. (E) Green colored excreta observed on day 3 of feeding pea aphids with green beans immersed in solution of water and green food coloring.

Next we tested the susceptibility of vegetable mediated delivery in other hemipteran insects such as the harlequin bug (*Murgantia histrionica*) (HB) and pea aphids (*A*. *pisum)* both of which are known insect pests. A similar approach (Fig 1) was taken to feed these insects with a solution containing green food coloring mediated through green beans. Observations revealed that both these insects were capable of surviving on the green bean (Figs 6 & 7). This was evident from the green excreta evidence of dietary ingestion from green beans (Fig 6G & 6H; Fig 7E). These results suggest that our newly developed vegetable delivery protocol has potential to be successful for other insects to deliver treatments such as dsRNA(s) for insect biocontrol.

**Fig 8:**
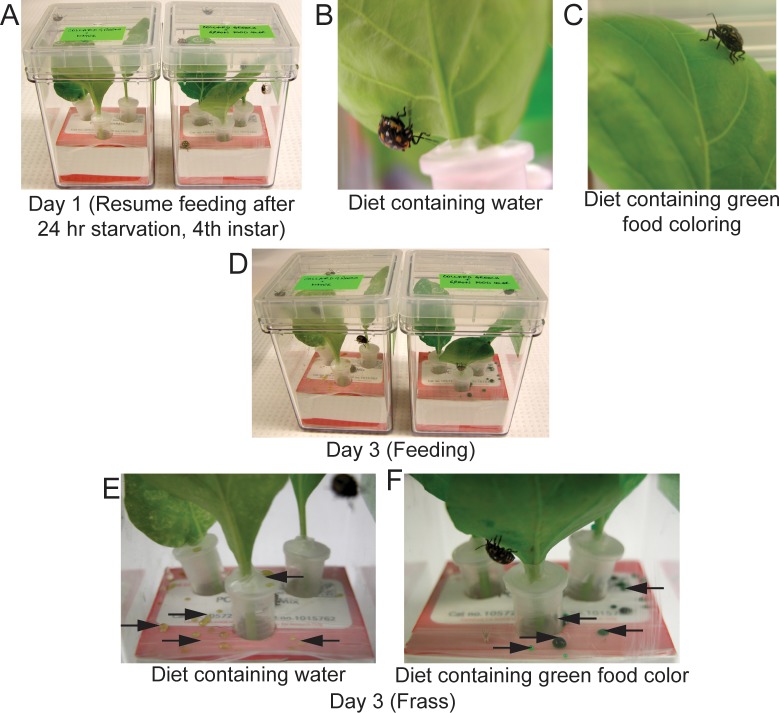
Harlequin bug (*M*. *histrionica*) feeding on baby collard greens. (A) Organically grown baby collard greens were washed with sodium hypochlorite. The petioles of these leaves were then immersed in ddH_2_O or ddH_2_O solution with green food coloring for a period of 3 hrs. Three each of 4^th^ instar HB nymph were allowed to resume feeding on these collard greens after 24 hr starvation in each magenta vessel. (B) HB feeding bioassay day 1. (C) HB feeding bioassay day 1 containing ddH_2_O solution with green food coloring, (D) Day 3 of feeding. (E) Excreta was observed on day 3 of feeding HB with collard greens immersed in water. (F) Green excreta was observed on day 3 of feeding HB with collard greens immersed in solution of water and green food coloring.

Additionally, delivery was tested using collard greens (*Brassica oleracea var*. *viridis*) to HB (Fig 8A) similarly as described for BMSB. Harlequin bugs are a known pest of cole crops such as crucifers or brassicas and infestation causes white stipples due to its piercing/sucking mode of feeding. Efficient uptake of both ddH_2_O and a solution of ddH_2_O with green food coloring by the HB were apparent from the green colored excreta observed on day 3 of the feeding assay (Fig 8D, 8E and 8F).

## Discussion

RNAi has emerged as a potential tool in functional gene regulation that may be of great importance in future years for the control of insect pests [51,52]. Microinjection and soaking methods utilized for dsRNA delivery have been documented to produce an effective RNAi response but have most commonly been used for fundamental studies of RNAi in insects. Efficient delivery of dsRNAs to insects for use as biopesticides in the environment requires a feasible, efficient and advantageous method, as demonstrated by topically applied dsRNA to citrus and grapevines [22,23]. RNAi biopesticide development is dependent upon the ability to deliver the specific dsRNA to the insect. Development of strategies which either use baits, sucrose solutions, yeast or bacteria for direct ingestion, or from topical application of dsRNA onto plants, or transgenic plants that can produce the dsRNA all have been shown to provide effective delivery [25,53].

This study successfully demonstrated the use of a vegetable, green bean, to deliver dsRNA designed to specifically impact and reduce an insect pest of global importance, BMSB. The selection of green beans as the vegetable for delivery relied on the ease of availability, cost, and natural attractiveness to the insect. Also evaluated were several artificial diets alongside the natural diets to identify the superiority of green beans as a suitable dsRNA delivery system for BMSB because they supported healthy growth of the animals under normal conditions. Similar experiments were conducted using rehydrating freeze-dried organic apples with dsRNA solution and by injecting organic raisins with a 10% sugar solution containing dsRNA, but these yielded limited success (data not shown). BMSB is a phloem-feeder causing damage by piercing and sucking from the vascular tissues of fruits and vegetables. The plant vascular system was suitable for uptake of *in vitro* synthesized dsRNA providing efficient delivery to the animal as demonstrated by reducing JHAMT and Vg gene expression in BMSB tissues.

This study also demonstrated the stability of JHAMT and Vg dsRNAs when using a vegetable-mediated delivery technique as verified by depletion in the level of targeted gene transcripts and the quantity of PCR product detected after 24 hr in the green beans. Degradation was measured using RT-PCR of total RNA from treated vegetables, which revealed rapid degradation of JHAMT and Vg dsRNA in green beans. This assay is particularly of significance because potential nucleic acids applied for the control of insect pests as either spray- or vegetable-mediated, will not persist long-term in plants or the environment [48]. RNAs are readily processed and degraded in prokaryotes by RNase III [54] while in eukaryotes multiple mechanisms exist that process dsRNAs [55].

RNAi could be successfully induced in the insect using this novel vegetable-mediated dsRNA delivery system. This delivery system could be used as a trapping system alongside BMSB attractants to keep BMSB away from higher-value crops or commodities. A similar method for treatment of soil of the plant’s root zone [22,23], and/or host plant seeds with dsRNA solution, could be used for delivery to insect pests through emerging plants. We also examined the vegetable-mediated delivery to other hemipteran insects such as the harlequin bug (HB) and pea aphid that displayed successful delivery using green beans. Although these pests do not prefer green beans they were observed to feed on them. This implies that specific vegetable mediated delivery systems such as dsRNA delivery to HB through collard greens may be used as a tool for inducing RNAi in HB. Potentially, selection of novel genes and modifications of vegetable-mediated dsRNA delivery methods may provide new strategies for deploying molecular biopesticides for a wide range of arthropod pests [17,28].

The advancement of RNAi-based technologies poses challenges that need to be addressed to make them more effective in coming years [52]. The slow progress in RNAi biopesticide development may be attributed to lack of efficient delivery methods and *in vivo* testing through methods of ingestion, efficiency of gene regulation by the RNAi machinery, durability of resistance and feasibility for application under field conditions [37]. These results demonstrate that gene-specific dsRNA can be delivered by oral feeding for RNAi to be induced in BMSB and other hemipteran insects. The oral RNAi delivery system described here makes possible the use of RNAi-mediated approaches in the environment for managing invasive insect pests. This oral delivery system may also be utilized to deliver RNA-guided genetic silencing systems that may be developed in the future to sap-feeding and chewing insects. The ability to trigger RNAi orally in such a highly specific manner in invasive insects that collectively are the biggest threat to global food security, provides a significant advancement towards safer ecological pest management.

## Materials and methods

### Artificial diets

Four different artificial diets were prepared as described:

1. *Artificial gypsy moth diet* was prepared by combining 120 g wheat germ, 10 g USDA vitamin Mix, 25 g casein, 8 g Wesson salts, 2.5 g sorbic acid, 1 g methyl paraben, 15 g agar and 825 ml water. The ingredients were added to a high-speed blender in boiling water, blended and poured into 96 well microtiter plates.
2. *2%* or *8% Artificial BMSB diet* was prepared by combining 10g agar (for 2% agar diet) or 40g (for 8% agar diet), 113g applesauce (Santacruz organic™), 50 ml organic apple juice, water to make up to 500 ml.
3. *Artificial BMSB diet with green bean puree* was prepared by combining 60 g wheat germ, 10 g USDA vitamin Mix, 8 g Wesson salts, 2.5 g Sorbic Acid, 1 g methyl paraben, 15 g Agar, 50 g cellulose, 200 g certified organic green beans (boiled and pureed), 75 g dextrose, 25 g sucrose, water to make up to 1 L.

These diets were poured into 96-well polypropylene plates, frozen overnight (-20°C), and placed in a Virtis Advantage Freeze Drier® (The Virtis, Gardiner, NY) for freeze drying. The frozen diets in 96-well plates were placed in the pre-frozen shelves -45°C and held for 20 min. The diets were further dried in the following steps under vacuum at 15 mTorr: -40°C for 600 min, -30°C for 420 min, -20°C for 300 min, -10°C for 300 min, 0°C for 60 min, 10°C for 60min, 20°C for 120 min, 30°C for 120 min, and 40°C for 120 min. The initial four steps are the primary drying phase and the last six steps are essential for secondary drying. The secondary drying steps are necessary to ensure the ability of the diet to absorb the treatment solution. The vacuum is released following completion of the freeze-drying program; the 96-well plates were removed and inverted to remove the diet pellets. These pellets were then placed in sterile plastic bags, and stored at 4°C prior to use.

### Vegetable diets

Two different vegetable diets were used:

1. Certified organic green beans (*Phaseolus vulgaris* L.) were washed with 0.2% sodium hypochlorite solution (J.T. Baker) for five minutes and later washed 3 times with ddH_2_O. The beans were trimmed from the calyx end to a total length of 7.5 cm. These beans were used as controls.
2. *Delivery of treatment through green beans*: Certified organic green beans were washed and trimmed as mentioned above. Next the beans were immersed in a capless 2 ml microcentrifuge tube containing a 300 μl solution containing 1:10 dilution of green food coloring (McCormick & Co., Inc, Hunt Valley, MD; ingredients: Water, Propylene Glycol, Fd&C Yellow 5, Fd&C Blue 1, and Propylparaben as preservative) or 5 μg (0.017 μg/μl) or 20 μg (0.067 μg/μl) of dsRNA in RNase DNase free water. Lean green beans were selected for this diet to ensure the beans fit in the 2 ml microcentrifuge tubes. To prevent any evaporation of the solution or animals entering the solution, the microcentrifuge tubes containing the beans were sealed with parafilm. These tubes were kept at room temperature for 3 hours allowing for the solution to rise to the style of the green bean through capillary action. The tubes were further placed in a small box to keep them upright and enclosed in magenta jars (Sigma).
3. *Brassica oleracea var*. *viridis (Collard greens) as delivery system for HB*: Young collard green leaves measuring about 7.5–8.5 cm in length were snipped at the petiole, washed and trimmed as mentioned above. Next the leaves were immersed at the petiole end in a cap less 2 ml microcentrifuge tube containing a 300 μl solution containing either RNase/DNase free water or 1:10 dilution of green food coloring (McCormick & Co., Inc, Hunt Valley, MD) in RNase DNase free water. To prevent any evaporation of the solution or animals entering the solution, the microcentrifuge tubes containing the leaves were then sealed with parafilm. These tubes were stored at room temperature for 3 hours allowing for the solution to rise to the leaf surface. The tubes were further placed in a small box to keep them upright and enclosed in magenta jars (Sigma-Aldrich, St. Louis, MO).

### Feeding assays

BMSB (*H*. *halys*) insects were reared at USDA-ARS in the Beltsville Agricultural Research Center, Beltsville, MD as previously described [56]. Briefly, insects were reared in ventilated plastic cylinders (21621 cm OD) on a diet of organic green beans, shelled sunflower and buckwheat seeds (2:1, w/w), and distilled water supplied in cotton-stopped shell vials. Eggs were collected weekly, hatched in plastic Petri dishes with a water vial. Once the animals molted to second-instars, nymphs were transferred to larger rearing cages until adulthood. Adults, males and females were separated 1 to 2 days post emergence, and subsequently maintained in different containers. Insects were maintained in Thermo Forma chambers (Thermo Fisher Scientific) at 25°C and 72% relative humidity, under a 16L:8D photoperiod.

Pea aphids (*A*. *pisum*) were purchased from eNASCO (Fort Atkinson, WI) containing one live bean seedling inoculated with live pea aphids at different stages. HB (*Murgantia histrionica*) insects were field collected (2016) and reared at USDA-ARS in the Beltsville Agricultural Research Center, Beltsville, MD on collard greens in a green house under a 16L:8D photoperiod.

For feeding assays, early 4^th^ instar BMSB nymphs were selected that were hatched from the same egg mass. The animals were starved for 24 hrs prior to feeding bioassays. The animals were treated in groups of three per magenta vessel containing three green beans, or three green beans with green food coloring or dsRNA solution, or 4 freeze dried pellets rehydrated with 0.5 ml of 10% sugar solution. The diets were replenished or changed every week.

### *In vitro* synthesis of double stranded RNA

Genes specific to BMSB were selected by examining comparative transcriptome profiles [19], and regions of interest for each gene selected that varied between 200 to 500 base pairs. Polymerase chain reaction (PCR) was performed to generate products from genomic DNA using specific oligonucleotides and purified using a QIAquick® PCR purification kit (Qiagen). This PCR amplified region was then used as template generate dsRNA required for RNAi in BMSB. The primers used for PCR contained the T7 promoter sequence (5’-GAA TTA ATA CGA CTC ACT ATA GGG AGA-3’). LacZ, a gene that encodes ß-galactosidase was amplified from the *E*.*coli* genomic DNA and served as a negative control (mock) for RNAi (all primers used are listed in S1 Table). The PCR-amplified DNA was purified using a QIAquick® PCR purification kit (Qiagen). *In vitro* transcription to yield dsRNA was performed by combining 250 mM HEPES pH 7.5, 32 mM magnesium chloride, 10 mM Dithiothreitol (DTT), 2 mM spermidine, 25 mM each of rNTPs, 0.25 units of SUPERase In RNase inhibitor (Life Technologies), and 0.5 μg PCR amplified DNA in a final volume of 20 μl were incubated at 37°C for 5 min. After 5 min, 1 μg T7 RNA polymerase was added to the reaction and further incubated at 37°C overnight.

The reactions were then centrifuged for 2 min at 13,000 rpm to pellet the magnesium pyrophosphate. The supernatant was transferred to a new microcentrifuge tube and was treated with 2 units of RNA Qualified DNase (RQ1) (Promega, Madison, WI) followed by incubation at 37°C for 30 min. The reaction mixture was extracted with an equal volume of phenol/chloroform/isoamyl alcohol (25:24:1) and centrifuged. The aqueous layer was extracted with chloroform. One-fifth-volume ammonium acetate (5 M ammonium acetate + 100 mM EDTA) and 3 volumes of chilled 100% ethanol were added to the resulting aqueous layer. After incubating on ice for 10 min, the dsRNA was precipitated, washed with 75% ethanol, resuspended in nuclease free water and stored in the freezer for use.

### Total RNA isolation and cDNA synthesis

To measure the level of gene expression in treated 4^th^ instar BMSBs, whole animals were homogenized individually using a micro-pestle subsequent to dsRNA treatment. Total RNA was isolated from the tissue samples by soaking and homogenizing in 1 ml volume of TRIzol (Invitrogen). Reverse transcriptase PCR was used to generate cDNA, 200 ng of total RNA was incubated with a 0.5 mM deoxynucleoside triphosphate mixture, 0.65 μM each oligo(dT)_16_ (Life Technologies), and random hexamers (Life Technologies) at 65°C for 5 min. A cDNA synthesis mixture containing 10 mM dithiothreitol (DTT), 100 units of Superscript Reverse Transcriptase III (Life Technologies), and 2 units of SUPERase™ In RNase inhibitor (Life Technologies) was then added to the total RNA mixture, which was incubated at 25°C for 5 min, 50°C for 50 min. The reaction was terminated by incubation at 70°C for 15 min and the resulting cDNA was stored at -20°C.

### Quantitative real-time PCR analysis

Levels of transcripts expressed were measured by quantitative realtime PCR (qPCR) using SYBR green PCR master mix from SensiMix SYBR® from Bioline (Taunton, MA). The reactions were performed on an Applied Biosystems 7500 real-time PCR system. Data were analyzed with ABI Prism sequence detection system software. All analysis was performed in the linear range of amplification. Standards were determined by serial dilution of the cDNA prepared from total RNA isolated from gut tissue of a normal animal and used as a reference standard for the quantification of cDNA produced from RNA. The BMSB 18s RNA was used as an internal standard to correct for differences in RNA recovery from tissues [19]. Statistical differences in normalized expression levels were analyzed by one-way ANOVA analysis.

The data was plotted using Kaleidagraph® (Synergy software). Primers used for qPCR analysis are as listed in S1 Table.

## Supporting information

S1 Table Oligonucleotide sequences for RNAi. (DOCX)

## Abbreviations

dsRNA: double stranded RNA
RNAi: RNA interference
BMSB: brown marmorated stink bug
HB: harlequin bug
JHAMT: Juvenile hormone acid O-methyltransferase
Vg: Vitellogenin
