# Supplementary material for: Double strand RNA delivery system for plant-sap-feeding insects
Source: PLoS One. 2017 Feb 9;12(2):e0171861. doi: 10.1371/journal.pone.0171861 (PMC5300277; doi:10.1371/journal.pone.0171861)
Supplement: S1 Table — (DOCX) [file pone.0171861.s001.docx]

**S1 Table. Oligonucleotide sequences for RNAi**

| **Potential *H. halys* target genes** | | |
| --- | --- | --- |
| Accession | Size | Gene Name/Homology |
| XP_014293026.1 | 491 | *Vitellogenin-A1-like (Vg)* (Possible isoforms: *vitellogenin-2-like* isoform X1 XP_014291483.1; *vitellogenin-2-like* isoform X2 XP_014291484.1). |
| XP_014290953.1 | 545 | *Juvenile hormone acid O-methyltransferase-like (JHAMT)* (Possible homolog: juvenile hormone acid O-methyltransferase XP_014283772.1). |
| **Primers** | | |
| **PCR** | | |
| Gene Name/Homology |  |  |
| *Vg* | BMSB Vitellog P2 F | CAATTTGATCCACCGACTGTT |
| *Vg* | BMSB Vitellog P2 R | CCGCATGAATCTTACTCTGGA |
| *JHAMT* | BMSB JH P1 F | GGATGCTTATGAATAATCCAG |
| *JHAMT* | BMSB JH P1 R | GTATAGGATTGCCATTTTGG |
| **T7 PCR** | | |
| *Vg* | T7 BMSB Vitellog P2 4263 F | GAATTAATACGACTCACTATAGGGAGACCAAAGTTGGAAGGGAATGA |
| *Vg* | T7 BMSB Vitellog P2 4753 R | GAATTAATACGACTCACTATAGGGAGACCGCATGAATCTTACTCTGGA |
| *JHAMT* | BMSB JH T7 P1 F | GAATTAATACGACTCACTATAGGGAGAGGATGCTTATGAATAATCCAG |
| *JHAMT* | BMSB JH T7 P1 R | GAATTAATACGACTCACTATAGGGAGAGTATAGGATTGCCATTTTGG |
| *LacZ* | T7 LacZ RNAi F | GAATTAATACGACTCACTATAGGGAGATGAAAGCTGGCTACAGGA |
| *LacZ* | T7 LacZ RNAi R | GAATTAATACGACTCACTATAGGGAGAGCAGGCTTCTGCTTCAAT |
| **qPCR** | | |
| *Vg* | RT Vitellog P2 F | TTGATAGTTGTTTGGATTTTGAAGGT |
| *Vg* | RT Vitellog P2 R | TCTTACTTGATCAGCGCTCAGAA |
| *JHAMT* | BMSB JH RT P1 F | AGGAAAACCCAAAATGGCAAT |
| *JHAMT* | BMSB JH RT P1 R | ATGTATTCTTCTTTTGGATCTTTTCTTGAG |
| *18S* | BMSB 18S F3 | ATGCCCCCGCCTGTCCTTATT |
| *18S* | BMSB 18S R3 | TGAAAGCAGCCTGAATAGTGG |
